# Medical Qualifications

**Published:** 1901-09-07

**Authors:** 


### MEDICAL QUALIFICATIONS.

THE following is a complete list of the bodies granting full qualifications, which are accepted by the General Medical Council as admitting to the Register :—

THE UNIVERSITY OF OXFORD.
THE UNIVERSITY OF CAMBRIDGE.
THE UNIVERSITY OF LONDON.
THE UNIVERSITY OF DURHAM.
THE VICTORIA UNIVERSITY.
THE UNIVERSITY OF BIRMINGHAM.
THE UNIVERSITY OF EDINBURGH.
THE UNIVERSITY OF GLASGOW.
THE UNIVERSITY OF ABERDEEN.
THE UNIVERSITY OF ST. ANDREWS.
THE UNIVERSITY OF DUBLIN.
THE ROYAL UNIVERSITY OF IRELAND.

All these Universities grant registerable medical degrees.

THE CONJOINT EXAMINING BOARD IN ENGLAND.

This board is formed by the Royal College of Physicians of London and the Royal College of Surgeons of England, and on passing its examinations the candidate obtains a diploma from each body and becomes L.R.C.P., M.R.C.S.

THE SCOTTISH COLLEGES.

The Royal College of Physicians of Edinburgh, the Royal College of Surgeons of Edinburgh, and the Faculty of Physicians and Surgeons of Glasgow have instituted a system of conjoint examinations, on passing which the candidate obtains the diplomas of each body and becomes L.R.C.P.Edin., L.R.C.S.Edin., and L.F.P.S.Glasg.

THE CONJOINT EXAMINING BOARD OF IRELAND.

The Royal College of Physicians of Ireland and the Royal College of Surgeons in Ireland have arranged a series of examinations, on passing which the candidate obtains the diploma of each body and becomes L.R.C.P.I., L.R.C.S.I.

THE SOCIETY OF APOTHECARIES OF LONDON.

This body grants a full qualification—the L.S.A.—the bearers of which are informed that they may style themselves "physician and surgeon."

THE APOTHECARIES' HALL OF IRELAND.

This body grants a full qualification.

In accordance with the Medical Acts, a body called the General Medical Council has been created whose duty it is among other things to keep a Register of all duly qualified practitioners. Only those on this Register are recognised by law, and anyone whose name is not on the Register, giving himself out to be a qualified practitioner, is liable to prosecution.

